# A Simple and Highly Effective Ligand System for the Copper(I)-Mediated Assembly of Rotaxanes**

**DOI:** 10.1002/anie.201407817

**Published:** 2014-10-14

**Authors:** Christopher J Campbell, David A Leigh, Inigo J Vitorica-Yrezabal, Steffen L Woltering

**Affiliations:** School of Chemistry, University of ManchesterOxford Road, Manchester M13 9PL (UK); School of Chemistry, University of EdinburghThe King's Buildings, West Mains Road, Edinburgh EH9 3JJ (UK)

**Keywords:** copper, imines, rotaxanes, self-assembly, supramolecular chemistry

## Abstract

A [2]rotaxane was produced through the assembly of a picolinaldehyde, an amine, and a bipyridine macrocycle around a Cu^I^ template by imine bond formation in close-to-quantitative yield. An analogous [3]rotaxane is obtained in excellent yield by replacing the amine with a diamine, thus showing the suitability of the system for the construction of higher order interlocked structures. The rotaxanes are formed within a few minutes simply through mixing the components in solution at room temperature and they can be isolated through removal of the solvent or precipitation.

The Copper(I) template system based on 2,9-diphenyl-1,10-phenanthroline (dpp) ligands,[1] introduced by Sauvage and co-workers in the 1980s,[2] revolutionized the synthesis of catenanes, rotaxanes, and knots[3] and has remained a mainstay of interlocked molecule synthesis for three decades.[4] In the early years, the covalent capture of the threaded intermediates was often carried out through Williamson ether synthesis,[2,5] but ring-closing olefin metathesis[6] and CuAAC ‘click’ chemistry[7] have proved to be effective methods of choice in recent times.[3] Imine bond formation is a particularly attractive reaction for preparing interlocked molecules;[8] it occurs under mild conditions and its reversible nature allows ‘error-checking’ during the assembly process, particularly when combined with metal ion coordination.[9] Herein, we describe a ligand set featuring a bipyridyl macrocycle and a 2-pyridylimine axle assembled around Cu^I^ to form threaded molecular structures in close-to-quantitative yields.[10,11] The system is a simple-to-access, high yielding version of the traditional Sauvage interlocking ligand system.

Upon mixing equimolar quantities of solutions of bipyridyl macrocycle **1**, picolinaldehyde **2**, amine **3**, and Cu(MeCN)_4_PF_6_ in MeCN at room temperature (Scheme 1), the solution immediately turns dark red. ^1^H NMR spectroscopy (Figure 1 b) and mass spectrometry (see the Supporting Information) of the reaction mixture indicate that a single species, [2]rotaxane **4**, is assembled almost quantitatively within five minutes at room temperature (significantly faster than imine-based rotaxane formation with octahedral metal templates[8d]). The use of other solvents, including CH_2_Cl_2_, CHCl_3_, DMF, MeOH, and THF, also give high (typically >80 %) yields of **4**, but in these cases, the non-interlocked thread **5** and/or the starting materials are also observed in the crude reaction mixture. The reason that the yields are so high for an assembly process that features bidentate ligand motifs on each component is that the rotaxane is the only possible product that allows maximal site occupancy[2a,^12^] with a strict equimolar ratio of ligands and metal ion, and the reaction is carried out under thermodynamic control.

**Figure 1:**
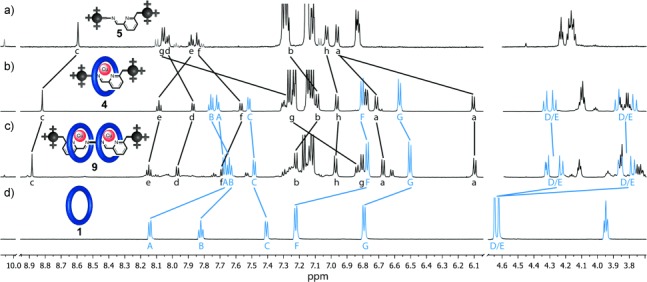
Partial ^1^H NMR spectra (600 MHz, CD_3_CN, 298 K) for: a) imine thread 5; b) [2]rotaxane 4; c) [3]rotaxane 9; and d) macrocycle 1. Signals from traces of aldehyde 2 in (a) are colored gray. The spectra for 4, 5, and 9 were measured from the reactions shown in Scheme 1, carried out in CD_3_CN with no work up, purification, or isolation procedures.

**Scheme 1:**
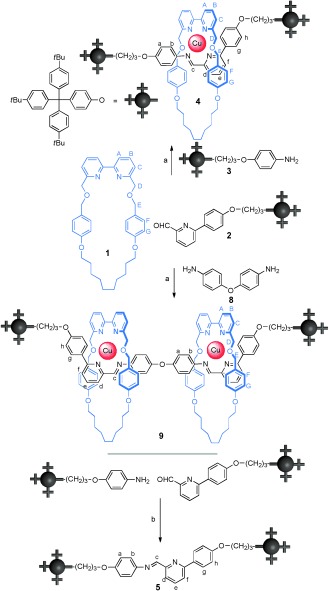
Cu^I^-mediated assembly of [2]rotaxane 4, [3]rotaxane 9, and imine thread 5. a) Cu(MeCN)_4_PF_6_, MeCN, RT, 5 mins, 96 % (4); 90 % (9). b) MgSO_4_, CH_2_Cl_2_, 318 K, 48 h, 40 %.

The threaded structure of [2]rotaxane **4** is apparent from comparison of its ^1^H NMR spectrum (Figure 1 b) with that of the non-interlocked thread **5** (Figure 1 a) and macrocycle **1** (Figure 1 d). As well as shielding of several of the thread protons (e.g. H_a_, H_d_, H_f_, H_g_, H_h_) in the rotaxane by aromatic rings of the macrocycle, the splitting of the macrocycle H_D_ and H_E_ methylene groups into AB systems (Figure 1 b) occurs as a consequence of the macrocycle being threaded by an unsymmetrical axle. The metal template could be removed by treating rotaxane **4** with Bu_4_NCN in CHCl_3_ to yield the metal-free imine rotaxane in high yield (see the Supporting Information).[13]

Single crystals of the metalated pseudorotaxane derivative **6** (for synthesis, the see Supporting Information), in which the bulky stoppers were replaced by methyl groups, were grown by vapor diffusion of water into a methanol solution of the pseudorotaxane, and the solid-state structure was determined by X-ray diffraction (Figure 2 a).[14] The tetrahedral Cu^I^ ion positions the components in a very similar co-conformation to that suggested by ^1^H NMR spectrum of the structure in CD_3_CN, with the phenyl rings of the macrocycle shielding the imine region of the axle and the bipyridine rings face-on to (and shielding) the phenyl group attached to the pyridine ring. The X-ray crystal structure of the reduced form of the pseudorotaxane (**7**) is shown in Figure 2 b.[14] Although the tetrahedral coordination geometry of the Cu^I^ remains intact, the relative orientations of the threaded components is changed somewhat compared to the imine system, with the macrocycle circumscribing the pyridine ring of the axle such that the bipyridine rings are no longer able to π-stack with the thread. The reduced motif can be used to assemble rotaxanes under kinetic control through a threading-and-stoppering procedure (see the Supporting Information). The Cu^I^ ions can be removed from the reduced form of the rotaxanes through treatment with an NH_3_/EDTA solution (see the Supporting Information). Methods to directly reduce the imine rotaxanes to amine rotaxanes are currently being developed in our laboratory.

**Figure 2:**
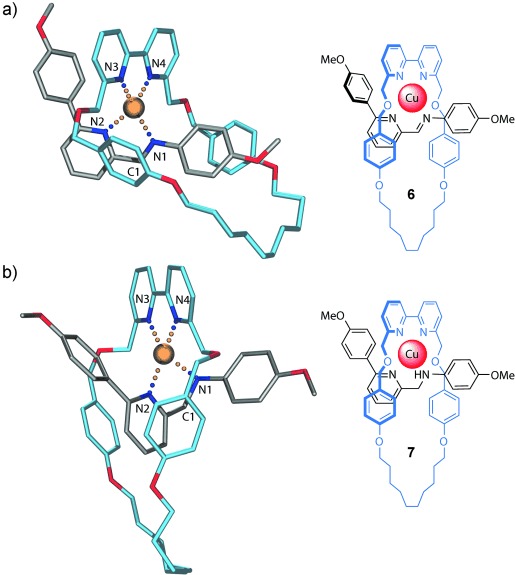
X-Ray crystal structures of the assembly motif in pseudorotaxanes 6 and 7.[14] a) Imine pseudorotaxane 6; selected bond lengths: *d*(Cu–N)=2.009–2.083 Å, *d*(C1–N1)=1.288 Å, N-Cu-N angles: 81.5–141.2°. b) Amine pseudorotaxane 7; selected bond lengths: *d*(Cu–N)=2.016–2.203 Å, *d*(C1–N1)=1.473 Å, N-Cu-N angles: 80.7–135.6°. Hydrogen atoms, counterions, and solvent molecules are omitted for clarity. Carbon atoms of the macrocycle are shown in light blue, carbon atoms of the threads gray; oxygen atoms red, nitrogen atoms dark blue, and copper atoms orange.

Replacing monoamine **3** with diamine **8** for the imine bond forming reaction led smoothly to the formation of [3]rotaxane **9** (Scheme 1). Again, the assembly of the threaded product is extremely efficient (90 % yield of isolated product). The ^1^H NMR spectrum of the [3]rotaxane reaction mixture prior to work up is shown in Figure 1 c, with only traces of aldehyde **2** (signal at 10.05 ppm) and the [2]rotaxane corresponding to one ring threaded onto the axle present in the crude reaction mixture (signal at 8.55 ppm).

In conclusion, we have discovered a simple and effective ligand set for the assembly of imine-based rotaxanes around copper(I) ions. The reactions proceed with high yield within a few minutes at ambient temperature and provide a readily accessible alternative to the classic Cu^I^–(dpp)_2_ rotaxanes. We anticipate that this facile method may find application in the synthesis of a range of interlocked molecular structures.
