# Supplementary material for: A Simple and Highly Effective Ligand System for the Copper(I)-Mediated Assembly of Rotaxanes
Source: Angew Chem Int Ed Engl. 2014 Oct 14;53(50):13771–4. doi: 10.1002/anie.201407817 (PMC4502974; doi:10.1002/anie.201407817)
Supplement: Supplementary file 1 [file anie0053-13771-sd1.pdf]

Angewandte Chemie
Eine Zeitschrift der Gesellschaft Deutscher Chemiker

Supporting Information

© Wiley-VCH 2014

69451 Weinheim, Germany

A Simple and Highly Effective Ligand System for the Copper(I)-Mediated Assembly of Rotaxanes**

Christopher J. Campbell, David A. Leigh, Inigo J. Vitorica-Yrezabal, and Steffen L. Woltering*

anie_201407817_sm_miscellaneous_information.pdf

1. General Experimental Section

All reagents and solvents were purchased from Sigma-Aldrich Chemicals and used without further purification. Dry solvents were obtained by passing through an activated alumina column on a Phoenix SDS solvent drying system (JC Meyer Solvent Systems, CA, USA). NMR spectra were recorded on a BrukerAvance III (equipped with a cryoprobe) instrument with an Oxford AS600 magnet or on a Bruker Avance 400 MHz instrument. Chemical shifts are reported in parts per million (ppm) from high to low frequency and referenced to the residual solvent resonance. Coupling constants (*J*) are reported in Hertz (Hz). Standard abbreviations indicating multiplicity were used as follows: s = singlet, d = doublet, t = triplet, q = quartet, quin = quintet, m = multiplet, br = broad. ^{1}H assignments were made using 2D NMR methods (COSY, HSQC, HMBC). Low resolution ESI mass spectrometry was performed with a Thermo Scientific LCQ Fleet or an Agilent Technologies 1200 LC system with 6130 single quadrupole MS detector mass spectrometer. Melting points (Mp) were determined using a Büchi Melting Point M-565 apparatus and are reported uncorrected. High resolution ESI (electrospray ionization) and EI (electron ionization) mass spectrometry were carried out by the mass spectrometry services at the University of Manchester or by the mass spectrometry services at the EPSRC National Mass Spectrometry Service Centre, Swansea, UK. High resolution NSI (nanospray ionization) and APCI (atmospheric-pressure chemical ionization) mass spectrometry were carried out by the mass spectrometry services at the EPSRC National Mass Spectrometry Service Centre, Swansea, UK.

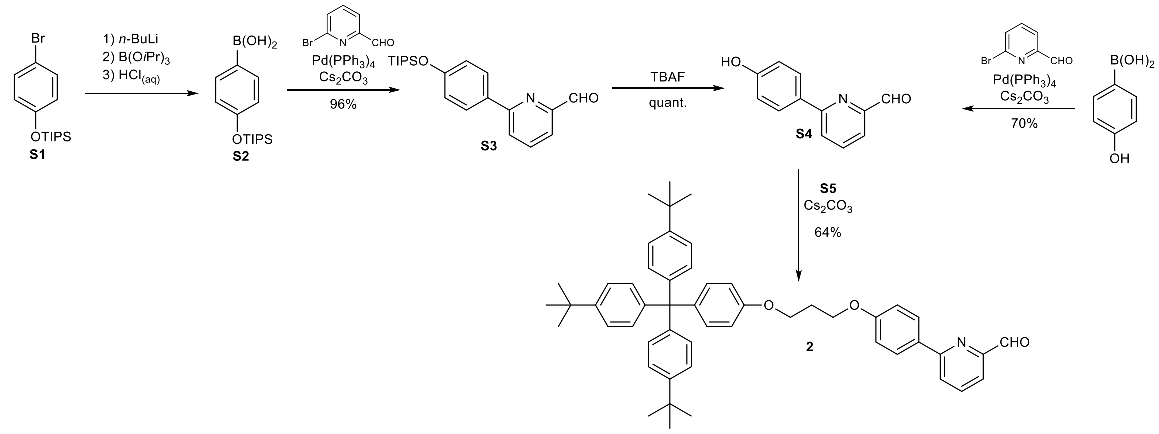

Scheme S1. Synthesis of aldehyde stopper **2**.

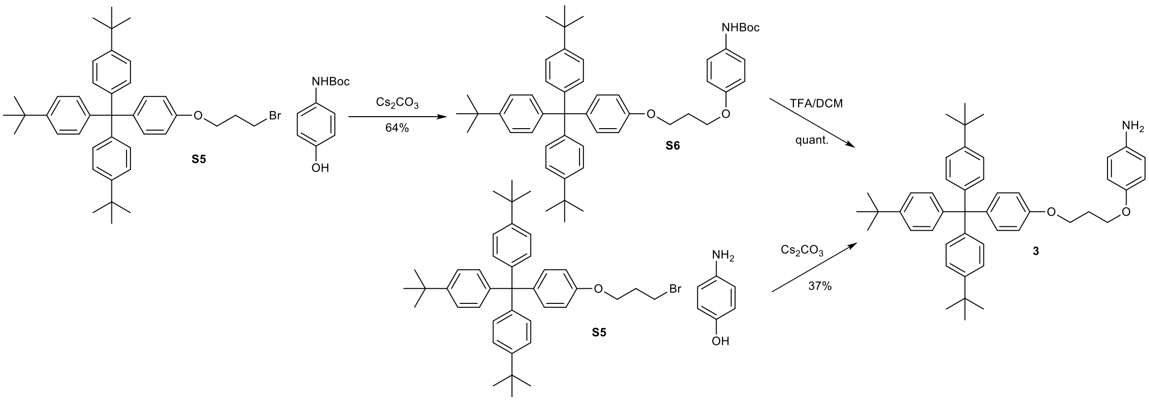

Scheme S2. Synthesis of aniline stopper **3**.

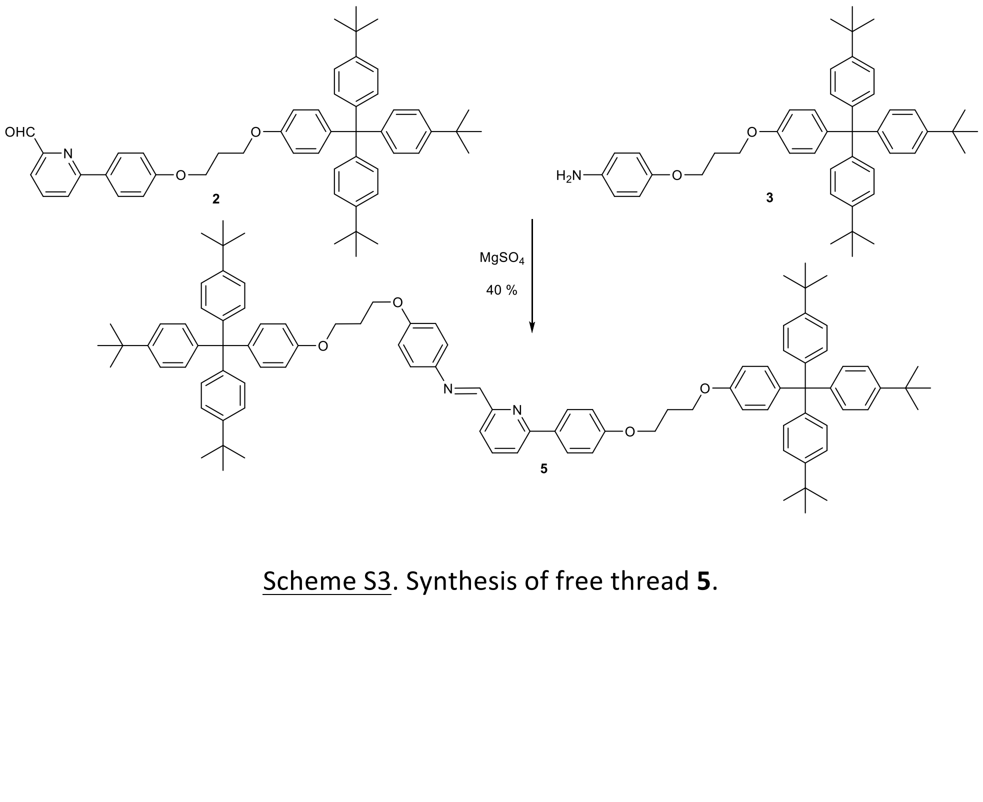

Scheme S3. Synthesis of free thread **5**.

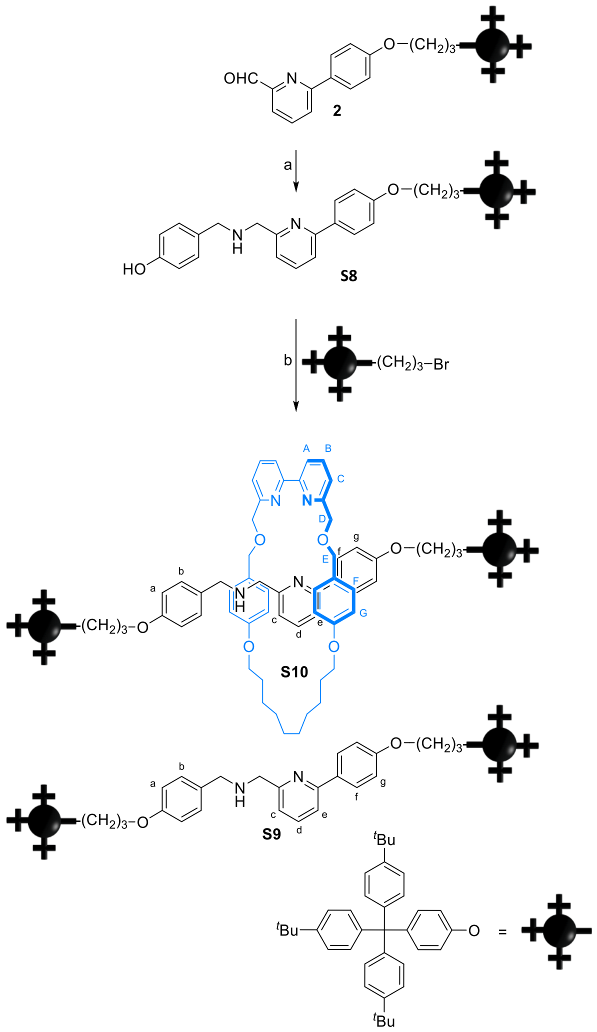

Scheme S4. Synthesis of demetallated rotaxane **S10** under kinetic control. Reagents and conditions: a) 4-aminophenol, $NaBH_3CN$, CH_2Cl_2/MeOH, 4.5 h, 71 %, b) **1**, $Cu(MeCN)_4PF_6$, Cs_2CO_3, DMF/CH_2Cl_2, rt to 50 °C, 48 h, NH_3/EDTA, 20 %.

The syntheses of macrocycle **1**[S1], **S1**[S2] and **S5**[S3] have been reported previously.

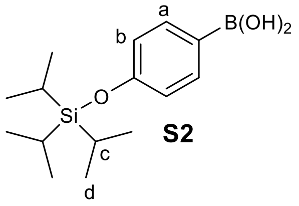

S2

S1 (1.5 g) was dissolved in THF (20 mL) under an inert atmosphere and cooled to -78 °C. *n*-BuLi (1.6 M solution in hexanes, 3.3 mL) was added and the mixture stirred at -78 °C for 30 min. An excess of B(O*i*Pr)$_3$ (5 mL) was added and the mixture was allowed to reach ambient temperature overnight under stirring. Aqueous HCl (2 N, 30 mL) was added and the mixture stirred at ambient temperature for 10 min. Ethyl acetate (50 mL) was added, the organic layer separated, and the aqueous phase washed with ethyl acetate (2 x 50 mL). The organic layers were combined and washed with sat. NaCl solution and dried with $MgSO_4$. Solvents were removed under reduced pressure to yield 1.3 g of a crude product which was used in the next step without further purification.

For an analytical sample, the crude product was purified by automated column chromatography (12 g silica, 100% CH_2Cl_2 → 10% EtOAc in CH_2Cl_2) to yield 0.7 g (52%) of **S2**.

M.p.: 220 °C; ^{1}H NMR (400 MHz, $CDCl_3$): δ = 8.10 (d, *J* = 8.4 Hz, 2H, H_a), 6.99 (d, *J* = 8.4 Hz, 2H, H_b), 1.31 (septet, *J* = 7.4 Hz, 3H, H_c), 1.13 (d, *J* = 7.4 Hz, 3H, H_d); ^{13}C NMR (100 MHz, $CDCl_3$): δ = 160.3, 137.6, 131.1, 119.8, 18.1, 12.9; LR-ESI-MS (negative mode): m/z = 293.1 [M-H]$^-$; HR-ESI-MS (TOF, negative mode): m/z =293.1758 [M-H]$^-$ (calc. for $C_{15}H_{26}O_3SiB$ 293.1749 [M-H]$^-$).

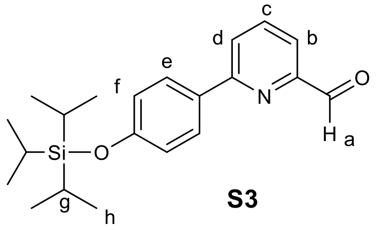

S3

6-Bromo-2-pyridinecarboxaldehyde (475 mg, 2.0 mmol) and boronic acid **S2** (750 mg, 2.5mmol) were dissolved in dioxane (18 mL) and water (2 mL) and flushed with nitrogen. Pd(PPh_3)$_4$ (230 mg, 0.2 mmol) and Cs_2CO_3 (3.26 g, 10 mmol) were added and the mixture stirred at 90 °C for 17 h. CH_2Cl_2 (100 mL) and water (100 mL) were added, the organic phase isolated and the aqueous phase washed with CH_2Cl_2 (2 x 50 mL). The combined organic layers were concentrated under reduced pressure and the crude product purified by automated column chromatography (12 g silica, 100% PE → 50% CH_2Cl_2 in PE) to yield 680 mg (96%) of **S3** as a yellow oil. R_f(CH_2Cl_2): 0.66; ^{1}H NMR (400 MHz, $CDCl_3$): δ = 10.15 (s, 1H, H_a), 8.00 (d, *J* = 8.8 Hz, 2H, H_e), 7.82-7.90 (m, 3H, H_b, H_c, H_d), 7.01 (d, *J* =

8.8 Hz, 2H, H_f), 1.30 (m, 3H, H_g), 1.13 (d, *J* = 7.4 Hz, 2H, H_h); ^{13}C NMR (100 MHz, $CDCl_3$): δ = 194.3, 158.0, 157.8, 152.7, 137.8, 131.1, 128.4, 123.9, 120.5, 119.2, 18.1, 12.8; LR-ESI-MS: m/z = 388.1 $[M+H+MeOH]^+$; HR-ESI-MS (TOF): m/z =356.2024 $[M+H]^+$ (calc. for $C_{21}H_{30}NO_2Si$ 356.2041 $[M+H]^+$).

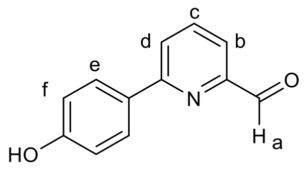

S4

<u>Method 1</u>

Aldehyde **S3** (178 mg, 0.5 mmol) was dissolved in THF (5 mL). TBAF (1 M solution in THF, 1 mL, 1.0 mmol) was added and the mixture was stirred at ambient temperature for 80 min. CH_2Cl_2 (100 mL) and water (100 mL) were added, the organic layer separated and the aqueous layer extracted with CH_2Cl_2 (2 x 50 mL). The combined organic layers were washed with sat. aqueous NaCl-solution and dried over $MgSO_4$. Removal of the solvents under reduced pressure yielded 105 mg of **S4** (quant.) as a yellow solid.

<u>Method 2</u>

6-Bromo-2-pyridinecarboxaldehyde (930 mg, 5 mmol) and *p*-Hydroxyphenylboronic acid (856 mg, 6.25 mmol were dissolved in dioxane (36 mL) and water (4 mL) and flushed with nitrogen. $Pd(PPh_3)_4$ (290 mg, 0.25 mmol) and Cs_2CO_3 (8.1 g, 25 mmol) were added and the mixture stirred at 90 °C overnight. CH_2Cl_2 (100 mL) and water (100 mL) were added, the organic phase isolated and the aqueous phase washed with CH_2Cl_2 (2 x 50 mL). The combined organic layers were washed with sat. aqueous NaCl-solution and the solvent removed under reduced pressure. The crude product was purified by automated column chromatography (24 g silica, 100% CH_2Cl_2 → 50% EtOAc in CH_2Cl_2) to yield 700 mg (70%) of **S4** as a yellow solid.

M.p.: 159 °C; ^1H NMR (600 MHz, $CDCl_3$): δ = 10.15 (s, 1H, H_a), 8.02 (d, *J* = 7.7 Hz, 2H, H_e), 7.83-7.91 (m, 3H, H_b, H_c, H_d), 6.98 (d, *J* = 7.7 Hz, 2H, H_f), 5.14 (s(br), 1H, OH); ^{13}C NMR (150 MHz, $CDCl_3$): δ = 194.3, 157.7, 157.2, 152.7, 137.9, 131.1, 128.8, 123.9, 119.3, 116.0; LR-ESI-MS: m/z = 200.1 $[M+H]^+$, 222.1 $[M+Na]^+$; HR-ESI-MS (TOF): m/z =200.0714 $[M+H]^+$ (calc. for $C_{12}H_{10}NO_2$ 200.0707 $[M+H]^+$).

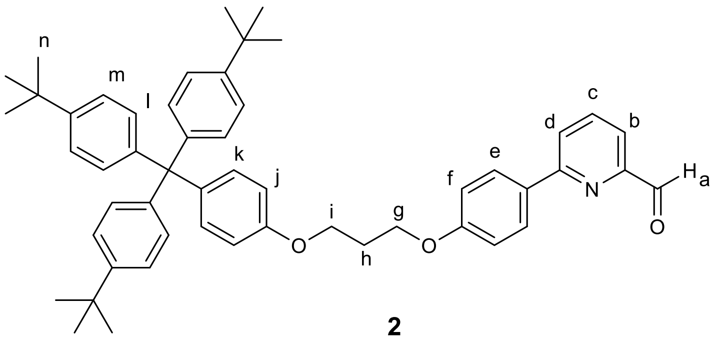

2

S4 (50 mg, 0.25 mmol) and **S5** (186 mg, 0.3 mmol) were dissolved in a mixture of DMF (10 mL) and CH_2Cl_2 (5 mL). Cs_2CO_3 (90 mg, 0.28 mmol) was added and the mixture stirred at ambient temperature for 24 h. CH_2Cl_2 (50 mL) and water (50 mL) were added and the organic layer isolated. The aqueous layer was washed with CH_2Cl_2 (2 x 25 mL) and the combined organic layers dried over $MgSO_4$. The solvents were removed under reduced pressure and the crude product purified by automated column chromatography (4 g silica, 100% PE → 50% CH_2Cl_2 in PE) to yield 120 mg (64%) of **2** as a colorless solid. $R_f(CH_2Cl_2)$: 0.74; M.p.: 224 °C; ^{1}H NMR (600 MHz, $CDCl_3$): δ = 10.16 (s, 1H, H_a), 8.05 (d, *J* = 8.0 Hz, 2H, H_e), 7.82-7.91 (m, 3H, H_b, H_c, H_d), 7.22 (d, *J* = 7.7 Hz, 6H, H_m), 7.06-7.10 (m, 8H, H_l, H_k) 7.04 (d, *J* = 8.0 Hz, 2H, H_f), 6.79 (d, *J* = 7.9 Hz, 2H, H_j), 4.26 (t, *J* = 6.0 Hz, 2H, H_g), 4.16 (t, *J* = 5.8 Hz, 2H, H_i), 2.29 (quint, *J* = 5.9 Hz, 2H, H_h), 1.29 (s, 27H, H_n);^{13}C NMR (150 MHz, $CDCl_3$): δ = 194.3, 160.5, 157.7, 156.7, 152.7, 148.4, 144.3, 139.9, 137.8, 132.4, 130.8, 130.8, 128.5, 124.2, 123.9, 119.2, 115.0, 113.1, 64.8, 64.2, 63.2, 34.4, 31.5, 29.5; LR-ESI-MS: m/z = 744.4 $[M+H]^+$, 766.5 $[M+Na]^+$; HR-ESI-MS (TOF): m/z =766.4238 $[M+Na]^+$ (calc. for $C_{52}H_{57}NO_3Na$ 766.4231 $[M+Na]^+$).

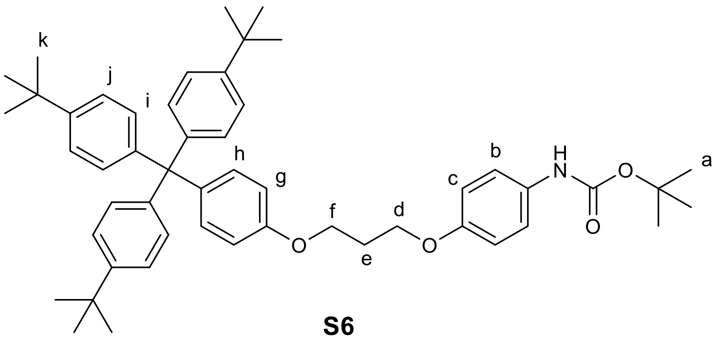

S6

N-Boc-4-aminophenol (105 mg, 0.50 mmol) and **S5** (156 mg, 0.25 mmol) were dissolved in DMF (10 mL). Cs_2CO_3 (90 mg, 0.28 mmol) was added and the mixture stirred at ambient temperature for 72 h. CH_2Cl_2 (50 mL) and water (50 mL) were added and the organic layer isolated. The aqueous layer was washed with CH_2Cl_2 (2 x 25 mL) and the combined organic layers dried over $MgSO_4$. The solvents were removed under reduced pressure and the crude product purified by automated column chromatography (4 g silica, 100% PE → 100% CH_2Cl_2) to yield 120 mg (64%) of **S6** as a

colorless solid. M.p.: 181 °C; ^{1}H NMR (600 MHz, $CDCl_3$): δ = 7.23 (d, *J* = 8.0 Hz, 8H, H_g, H_j), 7.09 (d, *J* = 8.0 Hz, 8H, H_h, H_i), 6.84 (d, *J* = 7.9 Hz, 8H, H_c), 6.77 (d, *J* = 7.9 Hz, 2H, H_b), 6.31 (s(br), 1H, NH) 4.08-4.15 (m, 4H, H_d, H_f), 2.22 (quint, *J* = 5.7 Hz, 2H, H_e), 1.50 (s, 9H, H_a), 1.29 (s, 27H, H_k); ^{13}C NMR (150 MHz, $CDCl_3$): δ = 156.8, 148.4, 144.3, 139.8, 132.4, 131.6, 130.9, 124.2, 130.9, 130.9, 124.2, 124.2, 115.0, 113.1, 65.0, 64.3, 63.2, 34.4, 31.5, 29.5, 28.5; LR-ESI-MS: m/z = 776.5 $[M+Na]^+$; HR-ESI-MS (TOF): m/z =776.4661 $[M+Na]^+$ (calc. for $C_{51}H_{63}NO_4Na$ 776.4650 $[M+Na]^+$).

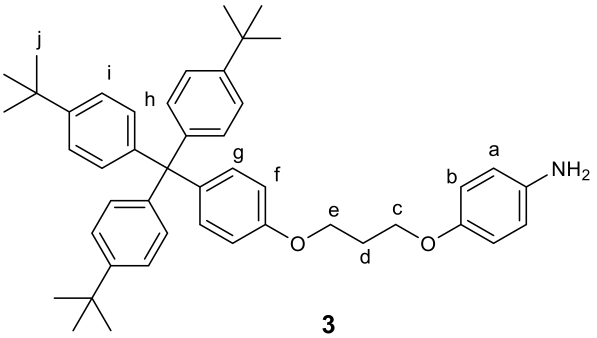

3

Method 1

4-Aminophenol (27 mg, 0.25 mmol) and **S5** (156 mg, 0.25 mmol) were dissolved in DMF (10 mL). Cs_2CO_3 (90 mg, 0.28 mmol) was added and the mixture stirred at ambient temperature for 36 h. CH_2Cl_2 (50 mL) and water (50 mL) were added and the organic layer isolated. The aqueous layer was washed with CH_2Cl_2 (2 x 25 mL) and the combined organic layers dried over $MgSO_4$. The solvents were removed under reduced pressure and the crude product purified by automated column chromatography (4 g silica, 100% PE → 100% CH_2Cl_2) to yield 60 mg (37%) of **3** as a red solid.

Method 2

S6 was dissolved in CH_2Cl_2 (2 mL) and TFA (0.5 mL) was added. The solution was allowed to stir at ambient temperature for 30 min. CH_2Cl_2 (50 mL) and sat. aqueous $NaHCO_3$ solution were added and the organic layer was isolated. Evaporation of the solvent afforded 59 mg (97%) of **3** as a red solid.

R_f(CH_2Cl_2): 0.22; M.p.: 195 °C; ^{1}H NMR (600 MHz, $CDCl_3$): δ = 7.23 (d, *J* = 8.6 Hz, 6H, H_i), 7.09 (d, *J* = 8.6 Hz, 6H, H_h), 7.08 (d, *J* = 9.1 Hz, 2H, H_g), 6.76 (d, *J* = 9.1 Hz, 2H, H_f), 6.75 (d, *J* = 8.8 Hz, 2H, H_b), 6.65 (d, *J* = 8.8 Hz, 2H, H_a), 4.12 (t, *J* = 6.1 Hz, 2H, H_e), 4.08 (t, *J* = 6.1 Hz, 2H, H_c), 3.53 (s(br), 2H, NH_2), 2.21 (quint, *J* = 6.1 Hz, 2H, H_d), 1.30 (s, 27H, H_j); ^{13}C NMR (150 MHz, $CDCl_3$): δ = 156.8, 152.4, 148.4, 144.3, 139.7, 139.6, 130.9, 130.9, 124.1, 116.8, 115.8, 113.1, 65.3, 64.5, 63.2, 34.4, 31.5, 29.6;
LR-ESI-MS: m/z = 654.5 $[M+H]^+$; HR-ESI-MS (TOF): m/z =676.4130 $[M+Na]^+$ (calc. for $C_{46}H_{55}NO_2Na$ 676.4126 $[M+Na]^+$).

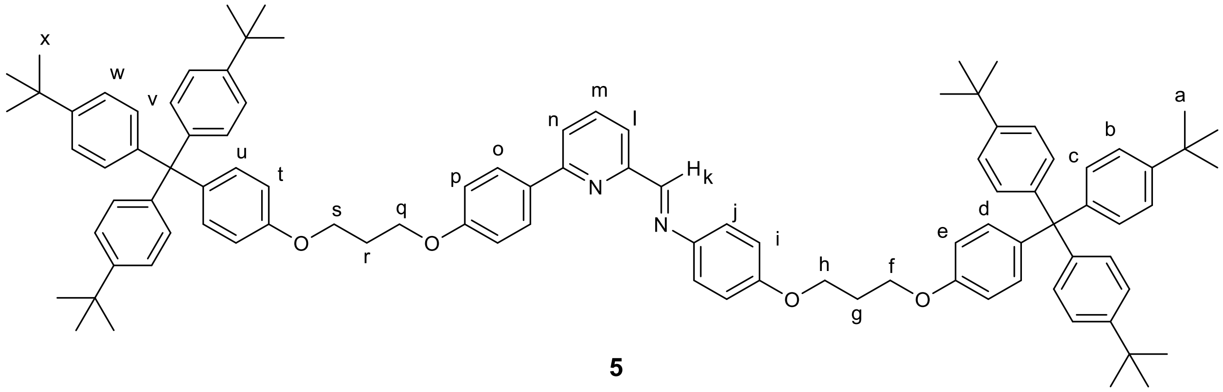

5

2 (74 mg, 100µmol) and **3** (65 mg, 100 µmol) were dissolved in CH_2Cl_2 (2 mL) and anhydrous $MgSO_4$ (100 mg) was added. The mixture was stirred for 2 d at 45 °C. The reaction mixture was purified by automated column chromatography (4 g silica, 100% PE → 100% CH_2Cl_2) to yield 55 mg (40 %) of **5** as a yellow solid.

M.p.: 164 °C; ^{1}H NMR (600 MHz, $CDCl_3$): δ = 8.72 (s, 1H, H_k), 8.12 (d, J = 7.8 Hz, 1H, H_l), 8.02 (d, J = 8.8 Hz, 2H, H_o), 7.81 (t, J = 7.8 Hz, 1H, H_m), 7.71 (d, J = 7.8 Hz, 1H, H_n), 7.35 (d, J = 8.7 Hz, 2H, H_j), 7.22-7.25 (m, 12H, H_b, H_w), 7.11 (d, J = 8.6 Hz, 16H, H_c, H_d, H_u, H_v), 7.03 (d, J = 8.8 Hz, 2H, H_p), 6.97 (d, J = 8.7 Hz, 2H, H_i), 6.81 (d, J = 9.0 Hz, 4H, H_e, H_t), 4.15-4.27 (m, 8H, H_f, H_h, H_q, H_s), 2.25-2.32 (m, 4H, H_g, H_r), 1.31 (s, 27H, H_a), 1.31 (s, 27H, H_x); ^{13}C NMR (150 MHz, $CDCl_3$): δ = 160.1, 159.3, 158.3, 157.0, 156.7, 156.7, 154.9, 148.4, 144.3, 143.9, 139.8, 139.8, 137.4, 132.4, 131.7, 130.9, 129.2, 128.4, 124.2, 122.9, 121.0, 119.0, 115.1, 114.9, 113.1, 64.9, 64.7, 64.3, 64.3, 63.2, 34.4, 31.5, 29.5.

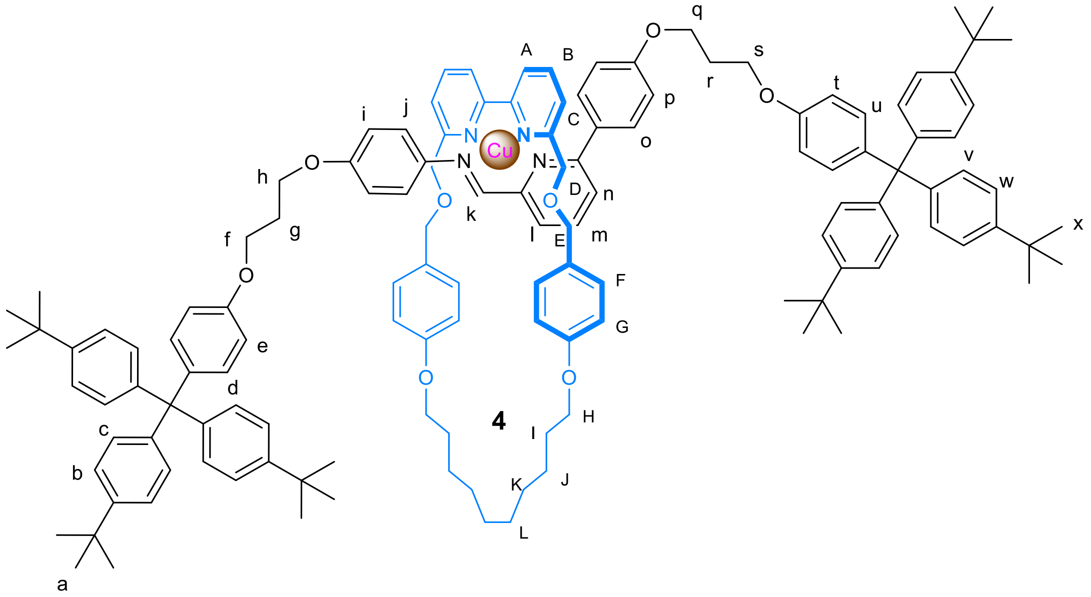

3 (32.7 mg, 50 µmol), **2** (37.2 mg, 50 µmol), **1** (28.3 mg, 50 µmol), and Cu(MeCN)$_4$PF$_6$ (18.6 mg, 50 µmol) were dissolved in MeCN (6 mL). The mixture was stirred for 5 minutes and the solvent removed under reduced pressure to yield 95 mg (96 %) of **4** as a dark red solid.

M.p. 178 °C; ^{1}H NMR (600 MHz, CD$_3$CN): δ = 8.82 (s, 1H, H$_k$), 8.08 (t, J = 7.8 Hz, 1H, H$_m$), 7.87 (d, J = 7.5 Hz, 1H, H$_l$), 7.76 (t, J = 7.8 Hz, 2H, H$_B$), 7.72 (d, J = 7.8 Hz, 2H, H$_A$), 7.57 (d, J = 7.7 Hz, 1H, H$_n$), 7.52 (d, J = 7.5 Hz, 2H, H$_C$), 7.27 (d, J = 8.6 Hz, 6H, H$_b$ or H$_w$), 7.23 (d, J = 8.7 Hz, 8H, H$_b$ or H$_w$, H$_o$), 7.08-7.16 (m, 18H, H$_j$, H$_c$, H$_d$, H$_u$, H$_v$), 6.97 (d, J = 8.9 Hz, 2H, H$_p$), 6.81 (d, J = 8.5 Hz, 4H, H$_F$), 6.77-6.79 (m, J = Hz, 4H, H$_e$, H$_t$), 6.72 (d, J = 9.0 Hz, 1H, H$_i$), 6.57 (d, J = 56 Hz, 4H, H$_G$), 6.11 (d, J = 6.11 Hz, 1H, H$_{i'}$), 4.33 (d, J = 12.4 Hz, 2H, H$_E$), 4.27 (d, J = 12.4 Hz, 2H, H$_{E'}$), 4.07-4.12 (m, 4H, H$_f$, H$_s$), 3.74-3.90 (m, 12H, H$_h$, H$_q$, H$_D$, H$_H$), 2.0-2.2 (m, 8H, H$_g$, H$_r$, H$_I$), 1.71-1.76 (m, 4H, H$_J$), 1.48-1.59 (m, 8H, H$_K$, H$_L$), 1.26 (s, 27H, H$_a$ or H$_x$), 1.21 (s, 27H, H$_a$ or H$_x$); ^{13}C NMR (150 MHz, CD$_3$CN) δ 160.7, 160.2, 159.7, 159.1, 158.2, 157.9, 157.7, 155.7, 151.9, 151.7, 149.4, 149.4, 145.5, 140.9, 140.6, 140.5, 139.7, 139.5, 132.9, 132.6, 132.5, 131.2, 131.2, 130.3, 129.6, 129.5, 127.5, 126.8, 125.4, 125.4, 125.0, 124.2, 121.2, 116.5, 116.0, 115.0, 114.3, 114.2, 114.0, 72.7, 70.6, 68.5, 65.7, 65.0, 64.8, 64.7, 34.9, 31.5, 30.4, 29.8, 29.7, 29.5, 29.3, 29.1, 26.3; LR-ESI-MS: m/z = 2008.2 [M+Cu]$^+$; HR-ESI-MS (TOF): m/z =2008.0841 [M+Cu]$^+$ (calc. for $C_{134}H_{152}CuN_4O_8$ 2008.0901 [M+Cu]$^+$).

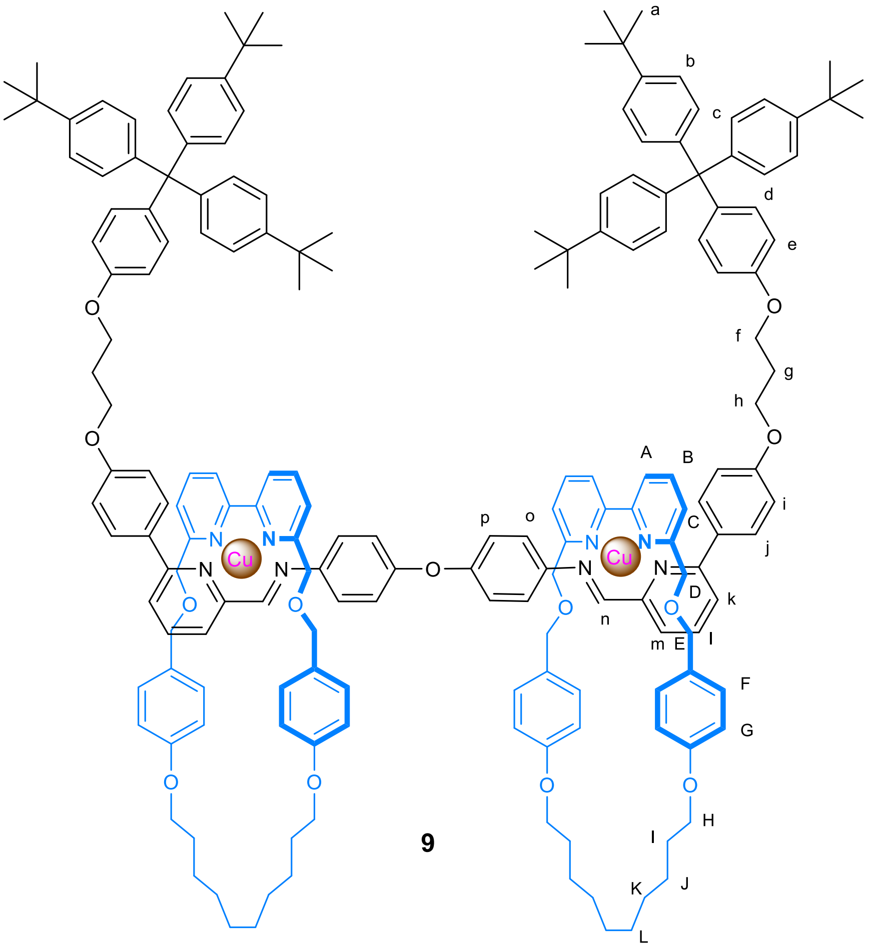

9

4,4'-Oxydianiline (4 mg, 20 µmol), **2** (30 mg, 40 µmol), **1** (23 mg, 40 µmol), and $Cu(MeCN)_4PF_6$ (15 mg, 40 µmol) were dissolved in MeCN (5 mL). The mixture was stirred for 5 minutes and then the product precipitated by adding water, to yield 60 mg (90 %) of **9** as a dark red solid.

m.p. 210 °C; ^{1}H NMR (600 MHz, CD_3CN): δ = 8.82 (s, 2H, H_n), 8.15 (t, *J* = 7.7 Hz, 2H, H_l), 7.98 (d, *J* = 7.6 Hz, 2H, H_m), 7.64-7.70 (m, 2H, 10H, H_k, H_A, H_B), 7.49 (d, *J* = 7.5 Hz, 4H, H_C), 7.22 (d, *J* = 8.9 Hz, 4H, H_o), 7.17 (d, *J* = 8.6 Hz, 16H, H_c, H_d), 7.12 (d, *J* = 8.6 Hz, 12H, H_b), 6.96 (d, *J* = 8.9 Hz, 4H, H_p), 6.81 (d, *J* = 8.4 Hz, 4H, H_j), 6.79 (d, *J* = Hz, 8H, H_F), 6.67 (d, *J* = 8.9 Hz, 4H, H_e), 6.67 (d, *J* = Hz, 2H, H_i), 6.51 (d, *J* = 8.5 Hz, 8H, H_G), 6.10 (d, *J* = 2 Hz, 2H, $H_{i'}$), 4.31 (d, *J* = 12.3 Hz, 4H, H_E), 4.23 (d, *J* = Hz, 4H, $H_{E'}$), 4.11 (t, *J* = 5.5 Hz, 4H, H_f), 3.70-3.88 (m, 20H, H_h, H_H, H_D), 2.08 (d, *J* = 5.7 Hz, 4H, H_g), 1.58-1.68 (m, 8H, H_I), 1.36-1.44 (m, 8H, H_J), 1.30-1.34 (d, *J* = Hz, 8H, H_K), 1.24-1.27 (m, 8H, H_L), 1.15 (s, 54H, H_a); ^{13}C NMR (150 MHz, CD_3CN) δ 160.2, 159.7, 159.4, 158.2, 157.9, 157.5, 151.7, 151.6, 149.4, 149.3, 145.4, 143.1, 140.9, 139.8, 139.7, 132.9, 132.5, 131.2, 130.3, 129.5, 129.4, 128.1, 127.3, 125.4, 125.2, 124.2, 121.1, 120.3, 118.3, 114.9, 114.2, 114.00, 72.8, 70.7, 68.5, 64.7, 64.6, 34.9, 31.5, 29.7, 29.5, 29.3, 29.2, 26.3; LR-ESI-MS: m/z = 1455.9 $[M+2Cu]^{2+}$; HR-ESI-MS (TOF): m/z =1454.7128 $[M+2Cu]^{2+}$ (calc. for $C_{188}H_{206}Cu_2N_8O_{13}^{2+}$ 1454.7143.0901 $[M+2Cu]^{2+}$).

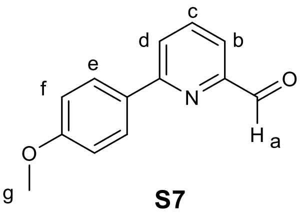

S7

Aldehyde **S4** (100 mg, 0.5 mmol) and K_2CO_3 (345 mg, 2.5 mmol) were suspended in DMF (3 mL). MeI (31 µl, 70 mg, 0.50 mmol) was added and the mixture stirred for 3 h at ambient temperature. The solvent was removed under reduced pressure and the residue purified by automated column chromatography (24 g silica, CH_2Cl_2 → 5% EtOAc in CH_2Cl_2) to yield 95 mg (89 %) of **S7** as a colorless solid.

m. p. 83 °C; ^{1}H NMR (600 MHz, $CDCl_3$): δ = 10.15 (s, 1H, H_a), 8.07 (d, *J* = 8.8 Hz, 2H, H_e), 7.83-7.91 (m, 3H, H_b, H_c, H_d), 7.04 (d, *J* = 8.8 Hz, 2H, H_f), 3.89 (s, 3H, H_g); ^{13}C NMR (150 MHz, $CDCl_3$) δ 194.3, 161.1, 157.7, 152.8, 137.8, 130.9, 128.5, 123.9, 119.2, 114.5, 55.6; LR-ESI-MS: m/z = 214.3 $[M+H]^+$; HR-ESI-MS (TOF): m/z =214.0872 $[M+H]^+$ (calc. for $C_{13}H_{12}NO_2$: 214.0868 $[M+H]^+$).

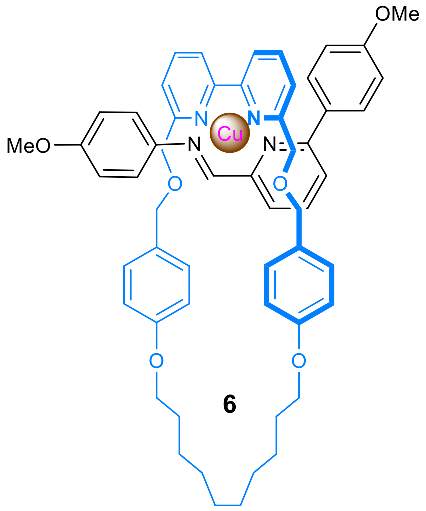

6

S7 (43 mg, 0.2 µmol), *p*-anisidine (25 mg, 0.2 µmol), **1** (113 mg, 0.2 mmol) and Cu(MeCN)$_4$PF$_6$ were dissolved in CH_2Cl_2 (2 mL) and the solution was stirred for 5 min at ambient temperature. The solvent was removed under reduced pressure to obtain a dark red solid. The crude product (5 mL) was dissolved in MeOH (1.25 mL) and filtered. Single crystals suitable for X-ray crystallography were obtained by vapor diffusion of water into a methanol solution of **6**.

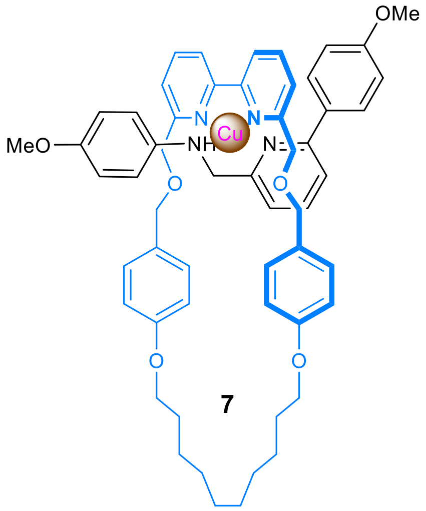

7

S7 (50 mg, 0.23 mmol) and *p*-anisidine (42 mg, 0.35 mmol) were dissolved in CH_2Cl_2 (5 mL) and MeOH (1 mL). NaBH$_4$ (9 mg, 0.23 mmol) was added and the resulting mixture stirred for 1 h at ambient temperature. The solvent was removed under reduced pressure and the residue filtered through a plug of silica (petrol ether → EtOAc). The solvent was removed under reduced pressure to obtain 10 mg of crude product. The crude, **1** (17 mg, 0.03 mmol) and Cu(MeCN)$_4$PF$_6$ (11 mg, 0.03 mmol) were dissolved in CH_2Cl_2 (5 mL), stirred for 5 min and the solvent removed under reduced pressure. 2.5 mg of the obtained product were dissolved in MeOH (750 µL) and filtered.

Single crystals suitable for X-ray crystallography were obtained by vapor diffusion of water into a methanol solution of **7**.

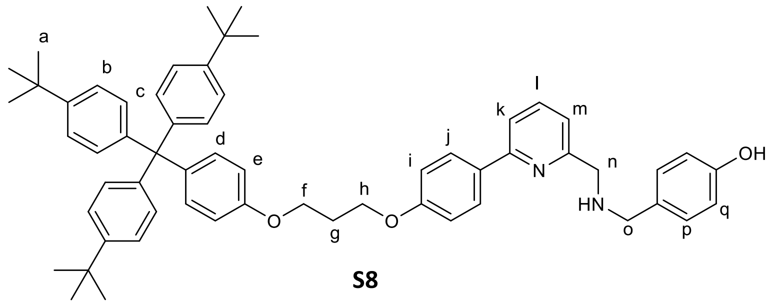

S8

Aldehyde stopper **2** (75 mg, 0.1 mmol) and 4-hydroxy-benzylamine (123 mg, 1.0 mmol) were dissolved in CH_2Cl_2 (18 mL) and MeOH (12 mL). $NaBH_3CN$ (63 mg, 1 mmol) was added and the mixture stirred at ambient temperature for 4.5 h. CH_2Cl_2 (25 mL) and sat. aqueous NaCl solution (25 mL) were added and the organic layer separated, filtered and concentrated under reduced pressure. The residue was dissolved in CH_2Cl_2 (20 mL), filtered and purified by automated column chromatography (24 g silica, 100% CH_2Cl_2 → 10% EtOAc in CH_2Cl_2) to yield 60 mg (71 %) of **S8** as a colorless solid. M.p. 131 °C; ^{1}H NMR (600 MHz, $CDCl_3$) δ 7.94 (d, *J* = 8.7 Hz, 2H, H_j), 7.66 (t, *J* = 7.7 Hz, 1H, H_l), 7.53 (d, *J* = 7.8 Hz, 1H, H_k), 7.22 (d, *J* = 8.5 Hz, 6H, H_b), 7.17 (d, *J* = 7.6 Hz, 1H, H_m), 7.13 (d, *J* = 8.3 Hz, 2H, H_p), 7.08 (d, *J* = 8.5 Hz, 8H, H_c, H_d), 6.98 (d, *J* = 8.7 Hz, 2H, H_i), 6.78 (d, *J* = 8.8 Hz, 2H, H_e), 6.64 (d, *J* = 8.3 Hz, 2H, H_q), 4.20 (t, 2H, H_h), 4.14 (t, 2H, H_f), 3.97 (s, 2H, H_n), 3.77 (s, 2H, H_o), 2.27 (quint., *J* = 6.1 Hz, 2H_g), 1.29 (s, 27H, H_a); ^{13}C NMR (150 MHz, $CDCl_3$) δ 159.9, 158.8, 156.8, 156.7, 155.6, 148.4, 144.3, 139.8, 137.4, 132.4, 132.1, 131.0, 130.8, 128.4, 124.2, 120.2, 118.4, 115.7, 114.8, 113.1, 64.7, 64.3, 63.2, 54.0, 53.6, 53.0, 34.4, 31.5, 29.5; LR-ESI-MS: m/z = 851.7 $[M+H]^+$.

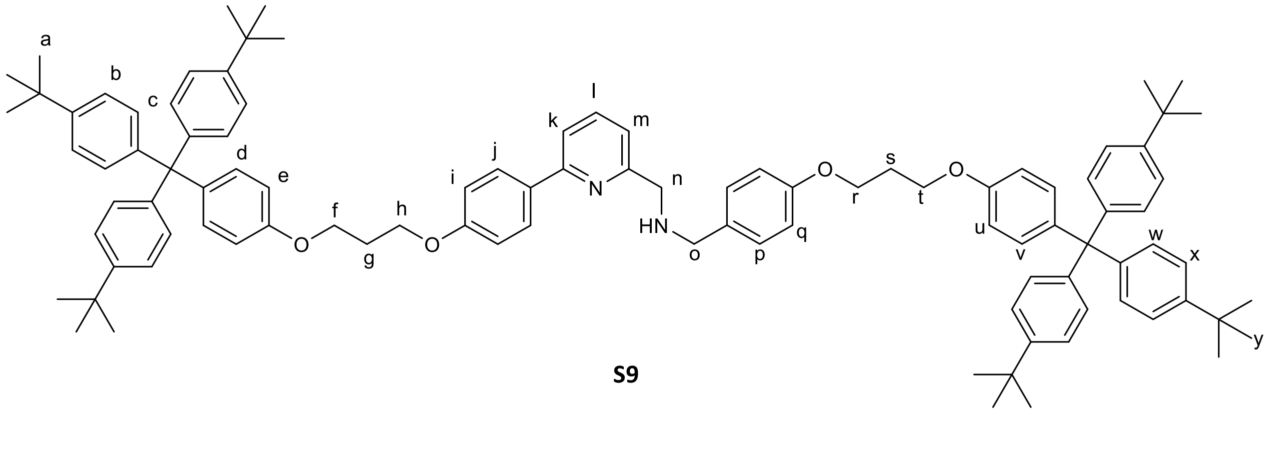

S9

A benzylamine stopper (45 mg, 67 µmol) and aldehyde stopper **2** (25 mg, 33 µmol) were dissolved in CH_2Cl_2 (3 mL). $MgSO_4$ was added and the resulting mixture stirred for 1 h at ambient temperature.

MeOH (0.3 mL) was added, followed by $NaBH_3CN$ (10 mg, 161 µmol), and stirring continued overnight. The mixture was filtered, the solvent removed under reduced pressure and the residue purified by automated column chromatography (4 g silica, CH_2Cl_2 → EtOAc) to yield 26 mg (58 %) of **S9** as a colorless solid. M.p. 166 °C; ^1H NMR (600 MHz, $CDCl_3$): δ = 7.87 (d, J = 8.7 Hz, 2H, H_j), 7.68 (t, J = 7.7 Hz, 1H, H_l), 7.57 (d, J = 8.1 Hz, 1H, H_k), 7.19-7.24 (m, 14 H, H_b, H_p, H_x), 7.05-7.10 (m, 16 H, H_c, H_d, H_v, H_w), 7.00-7.03 (m, 3 H, H_i, H_m), 6.79 (d, J = 8.7 Hz, 4H, H_e, H_u), 6.74 (d, J = 8.7 Hz, 2H, H_q), 6.01 (s(br), 1H, NH), 4.18-4.26 (m, 4 H, H_n, H_o, H_t), 4.16 (t, J = 5.7 Hz, 2H, H_r), 4.06-4.10 (m, 4H, H_f, H_h), 4.04 (dd, J_1 = 15.1 Hz, J_2 = 6.1 Hz, 1H, $H_{n'}$), 4.04 (dd, J_1 = 13.1 Hz, J_2 = 6.4 Hz, 1H, $H_{o'}$), 2.26-2.32 (m, 2H, H_g), 2.17-2.23 (m, 2H, H_s), 1.29 (s, 27H, H_a or H_y), 1.28 (s, 27H, H_a or H_y); ^{13}C NMR (150 MHz, $CDCl_3$): δ = 160.4, 159.6, 156.7, 156.7, 156.7, 151.8, 148.4, 148.4, 144.3, 144.2, 139.9, 139.9, 138.3, 132.4, 132.4, 131.5, 130.8, 130.8, 130.6, 128.3, 124.7, 124.2, 120.2, 119.4, 114.9, 114.9, 113.1, 113.0, 64.8, 64.6, 64.2, 64.1, 63.2, 63.2, 57.4, 56.1, 34.4, 34.4, 31.5, 31.5, 29.9, 29.4; LR-ESI-MS: m/z = 1395.6 $[M+H]^+$.

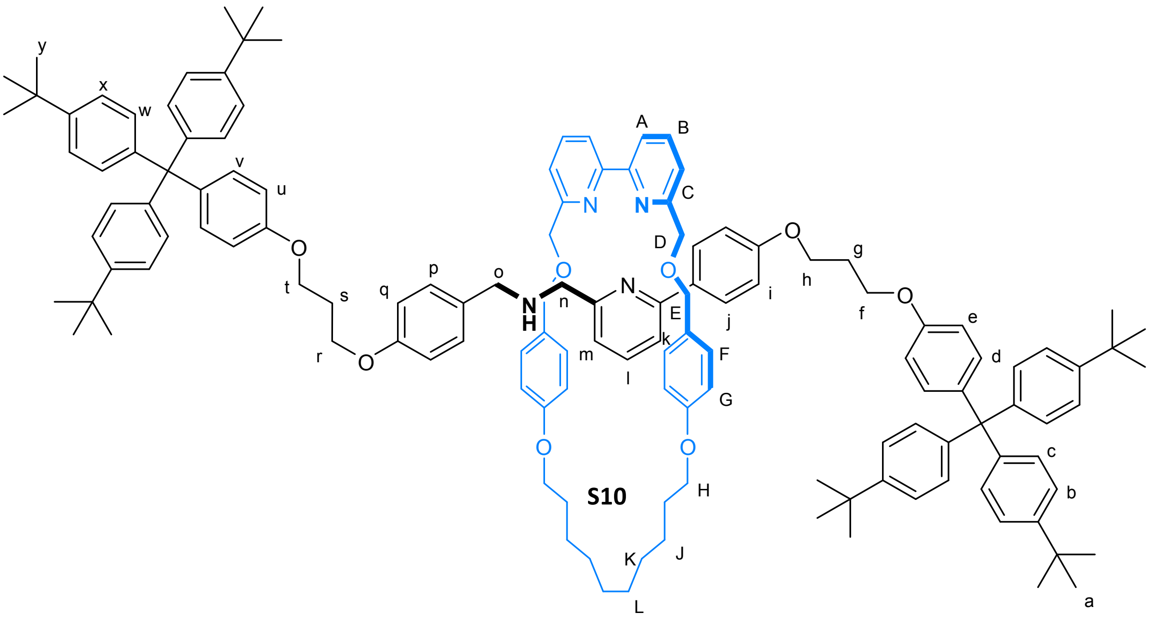

S8 (45 mg, 53 µmol), **S5** (30 mg, 53 µmol), **1** (130 mg, 208 µmol) and $Cu(MeCN)_4PF_6$ were dissolved in CH_2Cl_2 (5 mL) and stirred for 10 min at ambient temperature. The solvent was removed under reduced pressure and the residue redissolved in DMF (5 mL) and CH_2Cl_2 (1 mL). Cs_2CO_3 (32 mg, 100 µmol) was added and the resulting mixture stirred at 24 h at ambient temperature and then for 24 h at 50 °C. CH_2Cl_2 (20 mL) was added, the mixture filtered and the solvent removed under reduced pressure. The residue was dissolved in CH_2Cl_2 (100 mL) and washed with sat. aqueous EDTA solution containing ammonia (17 %) (2 x 50 mL). The solvent was removed under reduced pressure and the residue purified by automated column chromatography (4 g silica, CH_2Cl_2 → EtOAc) to yield 21 mg (20 %) of **S10** as a colorless solid.

m.p. 142 °C; ^{1}H NMR (600 MHz, $CDCl_3$): δ = 7.87 (d, J = 8.6 Hz, 2H, H_j), 7.62 (t, J = 7.6 Hz, 1H, H_l), 7.52 (d, J = 7.6 Hz, 1H, H_k), 7.39 (t, J = 7.7 Hz, 2H, H_B), 7.32 (d, J = 7.8 Hz, 2H, H_A), 7.22 (d, J = 8.6 Hz, 16H, H_b, H_F, H_x), 7.18 (d, J = 7.0 Hz, 1H, H_m), 7.08 (d, J = 8.5 Hz, 16H, H_c, H_d, H_v, H_w), 7.01 (d, J = 8.7 Hz, 2H, H_p), 6.93 (d, J = 8.6 Hz, 2H, H_i), 6.91 (d, J = 7.7 Hz, 2H, H_C), 6.84 (d, J = 8.3 Hz, 4H, H_G), 6.75-6.80 (m,4H, H_e, H_u), 6.55 (d, J = 6.7 Hz, 2H, H_q), 4.47-4.62 (m, 8H, H_D, H_E), 4.31 (t, J = 5.8 Hz, 2H, H_H), 4.04-4.22 (m, 12H, H_f, H_h, H_n,, H_o H_r, H_t), 3.72 (t, J = 5.9 Hz, 2H, $H_{H'}$), 2.11-2.30 (m, 8H, H_g, H_s, H_I), 1.89 (quint, J = 5.9 Hz, 4H, H_J), 1.29 (s, 54H, H_a, H_y), 1.25-1.26 (m, 8H, H_K, H_L); ^{13}C NMR (150 MHz, $CDCl_3$) δ 159.9, 159.9, 157.5, 157.1, 156.9, 156.7, 156.6, 156.4, 148.4, 144.3, 144.3, 139.8, 139.8, 137.5, 137.4, 132.4, 132.4, 132.2, 130.9, 130.9, 129.9, 129.1, 128.3, 128.2, 127.9, 125.0, 124.2, 124.2, 119.7, 118.4, 118.2, 117.9, 116.7, 114.7, 114.6, 114.1, 113.1, 113.0, 64.7, 64.6, 64.3, 64.3, 64.0, 63.1, 62.8, 62.5, 52.0, 51.3, 50.6, 50.0, 34.4, 32.1, 31.8, 31.5, 29.9, 29.5, 29.1, 22.9, 14.3; LR-ESI-MS: m/z = 1984.55 $[M+Na]^+$.

Isotope Pattern

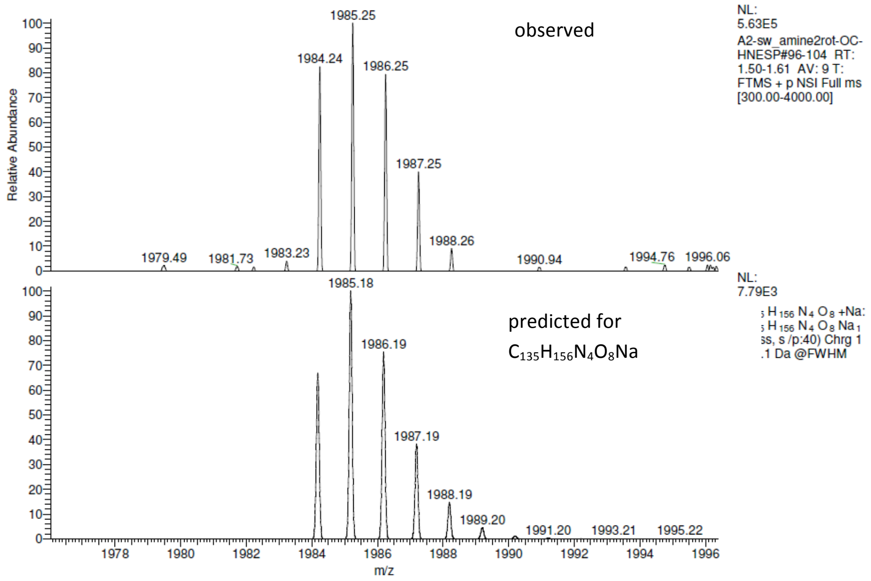

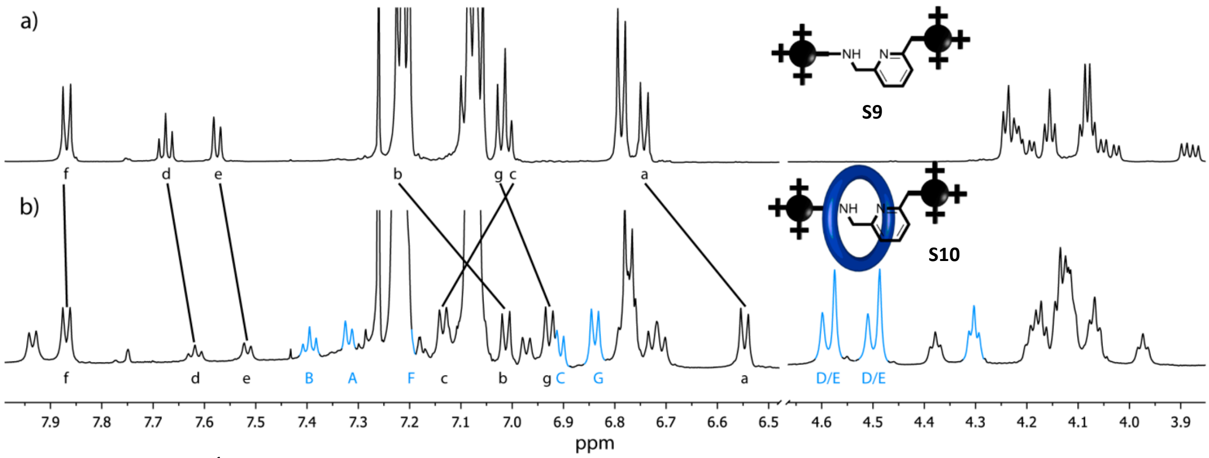

Figure S1. Partial ^{1}H NMR spectra (600 MHz, $CDCl_3$, 298 K) of: a) amine thread **S9**; b) demetallated amine [2]rotaxane **S10**. Signals originating from the macrocycle are shown in blue. The increased number of signals in the rotaxane is presumably due to rotaxane diastereomers arising from the presence of the asymmetric sp^3-nitrogen atom. Signals are numbered according to Scheme S4

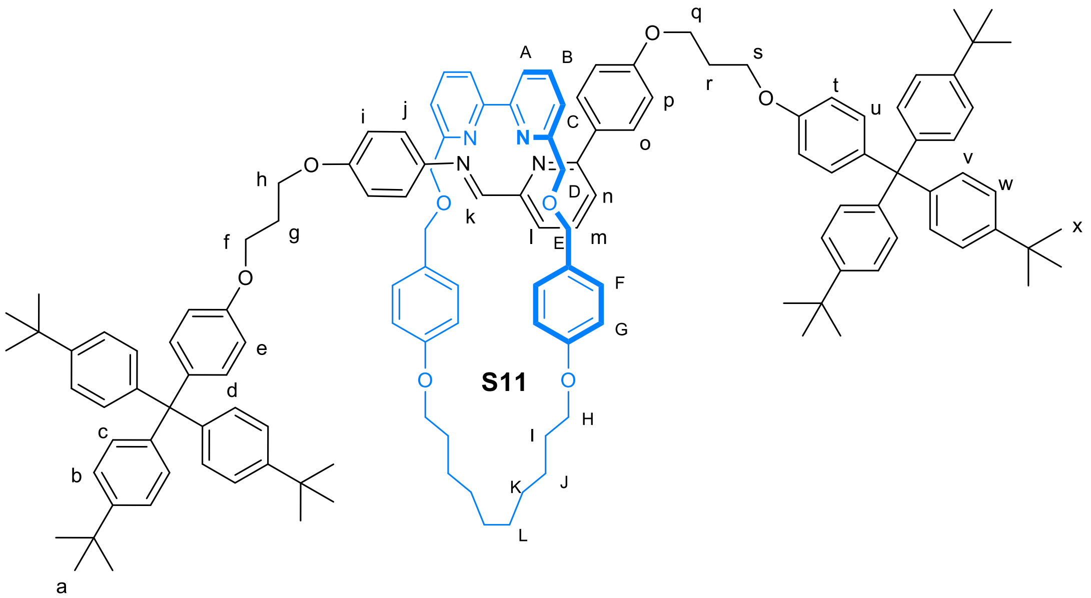

Rotaxane **4** (10 mg, 5 µmol) was dissolved in $CDCl_3$ (0.5 mL). NBu_4CN (10 mg, 37 µmol) was added. ESI-Mass spectrometry (m/z 1946.7) and ^{1}H NMR indicated formation of **S11** in high conversion (Figure S2b).

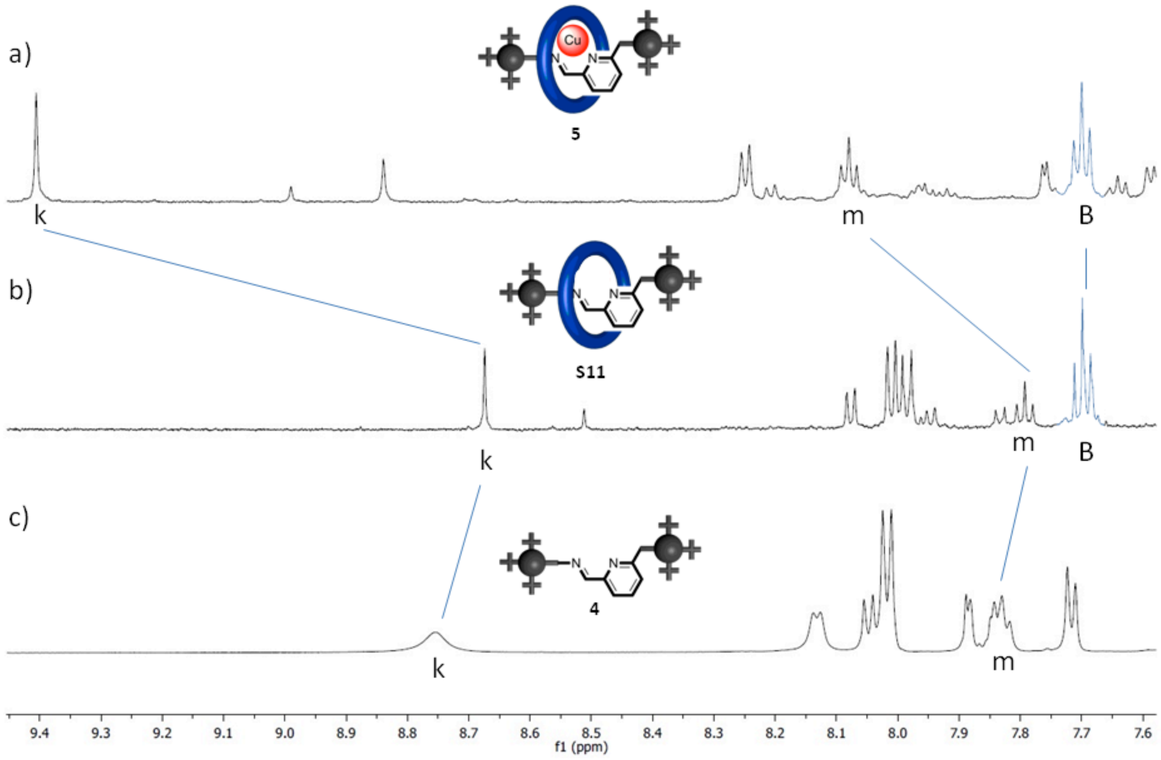

Figure S2. Partial ^{1}H NMR spectra (600 MHz, $CDCl_3$, 298 K) of: a) Copper imine [2]rotaxane **5**; b) demetallated imine [2]rotaxane **S11**; c) imine thread **4**. Signals originating from the macrocycle are shown in blue. Removal of the metal template shields the imine proton H_k by approx. 0.7 ppm.

References

[S1] a) J. Berná, J. D. Crowley, S. M. Goldup, K. D. Hänni, A.-L. Lee, D. A. Leigh, *Angew. Chem. Int. Ed.* **2007**, *46*, 5709-5713; b) T. Nakayama, M. Nomura, K. Haga, M. Ueda, *Bull. Chem. Soc. Jpn.* **1998**, *71*, 2979-2984.

[S2] P. Wipf, J. Methot, *Org. Lett.* **2000**, *2*, 4213-4216.

[S3] J. D. Crowley, K. D. Hänni, A.-L. Lee, D. A. Leigh, *J. Am. Chem. Soc.* **2007**, *129*, 12092-12093.
